# Efficacy of a Six-Month versus a 36-Month Regimen for Prevention of Tuberculosis in HIV-Infected Persons in India: A Randomized Clinical Trial

**DOI:** 10.1371/journal.pone.0047400

**Published:** 2012-12-14

**Authors:** Soumya Swaminathan, Pradeep Aravindan Menon, Narendran Gopalan, Venkatesan Perumal, Ramesh Kumar Santhanakrishnan, Ranjani Ramachandran, Ponnuraja Chinnaiyan, Sheik Iliayas, Padmapriyadarsini Chandrasekaran, Pooranaganga Devi Navaneethapandian, Thiruvalluvan Elangovan, Mai Tuyet Pho, Fraser Wares, Narayanan Paranji RamaIyengar

**Affiliations:** 1 National Institute for Research in Tuberculosis (Formerly Tuberculosis Research Centre), Indian Council of Medical Research, Chennai, India; 2 Division of Infectious Diseases, Beth Israel Deaconess Medical Center, Harvard Medical School, Boston, Massachusetts, United States of America; 3 Office of the WHO Representative to India, Delhi, India; London School of Hygiene and Tropical Medicine, United Kingdom

## Abstract

**Background:**

The optimal duration of preventive therapy for tuberculosis (TB) among HIV-infected persons in TB-endemic countries is unknown.

**Methods:**

An open-label randomized clinical trial was performed and analyzed for equivalence. Seven hundred and twelve HIV-infected, ART-naïve patients without active TB were randomized to receive either ethambutol 800 mg and isoniazid 300 mg daily for six-months (6EH) or isoniazid 300 mg daily for 36-months (36H). Drugs were dispensed fortnightly and adherence checked by home visits. Patients had chest radiograph, sputum smear and culture performed every six months, in addition to investigations if they developed symptoms. The primary endpoint was incident TB while secondary endpoints were all-cause mortality and adverse events. Survival analysis was performed on the modified intent to treat population (m-ITT) and rates compared.

**Findings:**

Tuberculosis developed in 22 (6.4%) of 344 subjects in the 6EH arm and 13 (3.8%) of 339 subjects in the 36H arm with incidence rates of 2.4/100py (95%CI- 1.4–3.5) and 1.6/100py (95% CI-0.8–3.0) with an adjusted rate ratio (aIRR) of 1.6 (0.8–3.2). Among TST-positive subjects, the aIRR of 6EH was 1.7 (0.6–4.3) compared to 36H, p = 0.8. All-cause mortality and toxicity were similar in the two arms. Among 15 patients with confirmed TB, 4 isolates were resistant to isoniazid and 2 were multidrug-resistant.

**Interpretation:**

Both regimens were similarly effective in preventing TB, when compared to historical incidence rates. However, there was a trend to lower TB incidence with 36H. There was no increase in isoniazid resistance compared to the expected rate in HIV-infected patients.

The trial is registered at ClinicalTrials.gov, NCT00351702.

## Introduction

Tuberculosis (TB) and Human Immunodeficiency Virus (HIV) are the two leading infectious causes of death globally, with TB being the most common cause of death among HIV-infected persons in the developing world [1], [2]. Although antiretroviral therapy (ART) reduces the risk of TB substantially, TB remains the most important cause of mortality and morbidity in patients on ART [3], [4]. India has a high burden of TB with an estimated prevalence of latent TB infection (LTBI) of 50%, an annual risk of TB infection of 1·5% and an estimated 1·96 million new cases of TB annually [5], [6]. Of the approximately 2·4 million people living with HIV in India, the incidence of TB has been reported to be as high as 6·9 cases/100 person-years (PY) [7], [8].

In the pre-ART era, several clinical trials demonstrated a reduction in TB incidence in HIV-infected patients with the administration of TB preventive therapy [9]–[11]. A recent meta-analysis found that isoniazid preventive therapy (IPT) reduces the risk of active TB by 33% overall and by 64% among adults with a positive tuberculin skin test (TST) [12]. The World Health Organization's (WHO) recommended regimen for TB preventive therapy in adolescents and adults living with HIV is isoniazid (H) 300 mg daily for six months [13]. Shorter regimens (e.g. two months of rifampicin and pyrazinamide) have been shown to be non-inferior, however rates of adverse effects were somewhat higher [14], [15]. While WHO recommended the use of TB preventive therapy for HIV-infected persons as early as 1998, very few national programmes have implemented this policy [16], [17]. Challenges faced by programmes in implementing IPT services include the difficulty in excluding active TB disease with certainty prior to initiation of IPT, poor adherence, potential emergence of drug resistance, uncertainty about the optimal length and composition of regimen and the cost-effectiveness of such an approach in high TB prevalence settings [18].

To determine the optimal duration of TB preventive therapy regimen in HIV-infected persons living in India, a TB-endemic country, we undertook a randomized clinical trial comparing six months of Isoniazid (H) and ethambutol (E) with 36 months (proxy for lifelong) of isoniazid. The rationale for the choice of isoniazid and ethambutol was to preserve rifampicin for chemotherapy and avoid the adverse effects of pyrazinamide whilst providing anti-tuberculosis activity in patients latently infected with isoniazid-resistant mycobacteria. While no previous studies have tested the EH combination, this was chosen in view of the 10–20% prevalence of isoniazid resistance among HIV-infected TB patients in India. [19], [20]. We hypothesized that the efficacy of a 6-month two drug combination (6EH) would be equivalent to using a single drug for a longer regimen (36H), with no more than 5% difference in cumulative (3-year) TB incidence between the two regimens, and that both would decrease TB incidence by at least 50% compared to historical data from the same setting [8].

## Methods

The protocol for this trial and supporting CONSORT checklist are available as supporting information; see Checklist S1 and Protocol S1.

### Ethics Statement

This study was approved by the Scientific Advisory Committee and Institutional Ethics Committee of Tuberculosis Research Centre, Chennai. A written informed consent was obtained from all the study participants before enrollment to the study.

### Study Design and Participants

This was a prospective, parallel arm, open label randomized controlled clinical trial conducted at the Tuberculosis Research Centre (TRC) clinics in Chennai and Madurai, southern India. Recruitment occurred between March 2001 and October 2005. HIV-infected individuals >18 years, without past or current evidence of TB disease, living within the defined area of intake, consenting to all the terms and conditions of the trial and fulfilling the laboratory criteria (normal chest radiograph, haemoglobin ≥70 g/L, granulocyte count ≥1·1×10^9^/L, platelet count ≥100×10^9^/L, serum alanine amino transferase ≤2·5 times the upper limit of normal, serum creatinine concentration <1·1 mg% and random plasma sugar <140 mg%) were enrolled.

### Randomisation and masking

Randomisation was performed using computer-generated random allocation sequences in permuted blocks of eight, stratified by TST status (< or >5 mm). The group assignment list was generated centrally before the start of the trial. Sequentially numbered, sealed, opaque envelopes containing the study group assigned were prepared independently and opened at the patient care facility at the time of allocation by a different group of statisticians.

### Procedures

Patients had a complete physical examination, sputum smear and culture (two overnight and one spot specimen), chest radiograph, tuberculin skin test (TST) and blood investigations. Sputum smears were examined by fluorescence microscopy, processed by the modified Petroff's method and cultured on Lowenstein-Jensen medium, with species identification and drug susceptibility testing [21]. Chest radiographs were read by two physicians independently and a third reader for discordant results. A TST was performed with 1TU PPD RT23 and read after 48–72 hours, with an induration of ≥5 mm considered positive. Additional baseline investigations included complete blood count (automated hematology analyzer ABX, France), CD4 count (FACSort flow cytometer, Becton Dickinson, USA), HIV viral load (Roche Amplicor automated viral load monitor, V1·5, Germany), renal and liver function tests, and random blood sugar (automated analyzer, Olympus Corporation, Tokyo, Japan). Urine was examined for albumin, sugar and acetyl isoniazid.

Regimens used were isoniazid 300 mg and ethambutol 800 mg daily for six months (6EH) or isoniazid 300 mg daily for 36 months (36H). Subjects in both study groups received 10 mg of pyridoxine daily during the treatment period and co-trimoxazole DS one tablet daily if CD4 count was <250 cells/mm^3^ for the entire period. From April 2004 onwards, patients were referred to the nearest government centre for evaluation and initiation of free ART if eligible (Stage III/IV disease or CD4 <250 cells/mm^3^).

Study medications were dispensed fortnightly for self-administration. During these visits, patients were asked to return the empty packets as well as any unused tablets. Patients expressing new symptoms were referred to the study physicians to rule out drug toxicity and/or active TB disease. Additionally, patients were monitored every three months for clinical status, adverse effects, adherence and the development of active TB disease. Details of adverse drug reactions and their management were recorded on a standardized toxicity form [22]. Adherence was assessed by pill counts of returned drugs, examination of spot urine samples for acetyl isoniazid and home visits done on random days approximately once in two weeks. Mycobacterial smear and culture (on two sputum specimens), chest radiograph, CD4 count, and liver and renal function tests were performed every six months and when clinically indicated. All patients were followed for 36 months post randomization.

### Endpoints

The primary endpoint was the development of pulmonary or extra-pulmonary TB during the study period. TB was classified as definite (positive mycobacterial culture) or probable (clinical/radiographic/histopathologic/biochemical features) based on review by a panel blinded to study assignment. Patients who developed active TB were treated with the standard national re-treatment regimen if they had received >1 month of study medications [23]. The secondary endpoints were all-cause mortality and adverse events. Cause of death was ascertained by verbal autopsy in case of death at home or hospital records in case of death in a health care facility and was classified as due to TB or to a non-TB cause.

### Statistical Analysis

The sample size was calculated anticipating a 50% reduction in TB incidence from historical data from a demographically similar cohort [8]. Assuming that the 36H regimen would reduce cumulative TB incidence over 3 years from 20% to 10%, an equivalence margin for 6EH of 5%, with a beta of 0·20, an alpha of 0·05, the number of patients required per arm was 325. This was increased by 10% to account for default/death making a final sample size of 350/arm. Data were analyzed using SPSS version 14. The primary analysis was a modified intent to treat analysis (m-ITT) excluding only those who had culture confirmed TB at baseline. Intent to treat analysis including all randomized patients and per-protocol analysis restricted to patients fulfilling all eligibility criteria, with treatment adherence of >80%, survival without TB beyond 6 weeks after randomization and with complete follow-up were also performed. The effect of treatment on the rate of TB was assessed using person-time from date of randomization until the earliest endpoint, i.e. documented TB disease or censoring (due to death, loss to follow-up or the end of the study at 36 months). Kaplan-Meier survival plots were used to calculate the crude effect of both regimens on TB-free survival and mortality and compared using the log rank test. Cox proportional hazards models were used to obtain an estimate of the effect of the regimens on the primary endpoint, after adjusting for potential confounders (sex, CD4 count and TST status) with ART as a time-dependent covariate. The main measures of effect used were rate ratios with confidence intervals.

The Accelerated Failure Time model was used to compare the curves wherever the hazards were found crossing. Equivalence was accepted if the point estimate and confidence interval was within the equivalence margin (5% around the cumulative 3-year incidence for 36H).

Berger-Exner test was performed taking the treatment response (development of active tuberculosis or not) as the dependent variable and the probability of predicting the concealed treatment assignment as well as the order of allocation within the block size of eight, as independent variables, along with other baseline covariates. The Berger-Exner test was used in conjunction with the comparison of baseline covariates, as the former was more sensitive to unobservable bias and the latter was more sensitive to observable selection bias [24].

Preliminary results were presented at the Conference on Retroviruses and Opportunistic Infections, February 2010, San Francisco [25]. The trial was registered in the NIH trial registry (NCT00351702).

## Results

**Figure 1:**
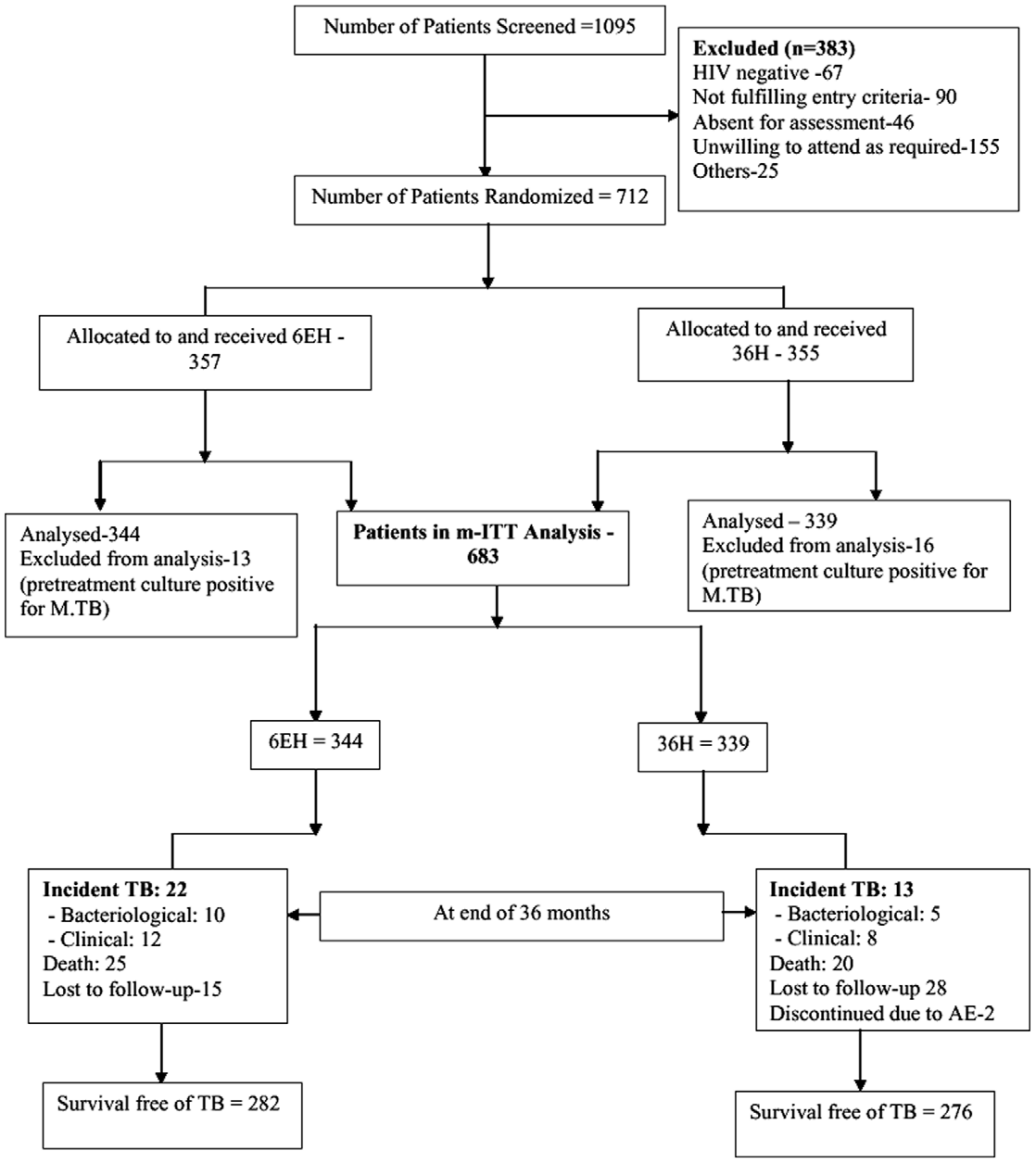
Trial Profile of all patients.

Of the 1,095 individuals screened, 712 were enrolled and randomized to the study regimens (ITT population), Figure 1. Berger Exner's showed that there was no prediction of outcome by the allocation probability within treatment groups ruling out selection bias. Twenty-nine patients were excluded because of positive *M.tuberculosis* baseline sputum cultures, leaving 683 patients in the modified (m-ITT) population. Baseline characteristics were similar in the two arms: majority of patients were <40 years of age, 63% were females and nearly half had a CD4 count >350 cells/mm^3^ (Table 1). Eighty eight percent (299/339) of patients in the 36H and 93% (320/344) in the 6EH arm comprised the per-protocol population (trial profile in supplementary appendix 1). The total follow-up period was 885 PY (median 2.6 years) in the 6EH arm and 844 PY (median 2.5 years) in the 36H arm. 169 patients started ART, mainly during the third year of follow-up – 76 patients in the 6EH arm accounting for 152 PY and 93 patients in 36H arm accounting for 172 PY of exposure (p = 0.4).

**Table 1:** Demographic Details of m-ITT Population (n = 683).

	6EH (n = 344)		36H (n = 339)	
Age (Mean ± SD), years	29·9±7		30·2±7	
Weight (Mean ± SD), kgs	51±10		50±10	
Females n (%)	218 (63)		212 (63)	
Age distribution	n	%	n	%
<25 years	106	30	97	29
25–40 years	208	61	216	64
>40 years	30	9	26	7
TST induration	n	%	n	%
<5 mm	203	59	207	61
5–10 mm	29	8	22	7
>10 mm	112	33	110	32
TST induration (Mean ± SD), mm	7·6	9·6	7·2	9·3
CD4 Count, Median (IQR),* cells/mm^3^	326 (208–520)		324 (197–463)	
CD4 count distribution	n	%	n	%
<100 cells/mm^3^	27	9	28	9
100–200 cells/mm^3^	51	16	56	18
201–350 cells/mm^3^	90	28	91	28
351–500 cells/mm^3^	62	20	68	21
>500 cells/mm^3^	86	27	77	24

* CD4 counts were available for 316 in 6EH and 320 patients in 36H arms respectively.

Tuberculosis developed in 22 (6.4%) of 344 subjects in the 6EH arm and 13 (3.8%) of 339 patients in the 36H arm with incidence rates of 2.4/100py (95% CI 1.4–3.5) and 1.6/100py (0.8–3.0) respectively; adjusted rate ratio 1.6 (95% CI 0.8–3.2). The two rates were equivalent statistically. Rates of TB incidence, death and adjusted incidence rate ratio (aIRR) for the total group and among TST positive and TST negative subjects are provided in Table no. 2 and 3 respectively. No statistically significant differences were observed between the two regimens by ITT, m-ITT or per-protocol analyses. Table 4 shows the TB incidence and death rates and rate ratios stratified by sex and CD4 count.

**Table 2:** Crude rates and adjusted incidence rate ratios for TB incidence and death, in ITT, m-ITT (excluding culture positive cases at baseline) and per-protocol population.

		TB			Death	
	Number of cases	Crude rate/100py (95% CI)	aIRR (95% CI)	Number of deaths	Crude Rate/100py (95% CI)	aIRR (95% CI)
**ITT**						
6EH (357)		2.33(1.36–3.31)	1.15(0.68–1.95)		2.65(1.61–3.69)	1.20(0.66–2.18)
36H (355)		1.39(0.63–2.14)	ref		2.13(1.20–3.07)	Ref
**m-ITT**						
6EH (344)	22	2·44(1.42–3.46)	1.59(0.79–3.21)	25	2·77(1.68–3.86)	1.22(0.65–2.29)
36H (339)	13	1·55(0.79–3.03)	1.0 (reference)	20	2·21(1.24–3.18)	1.0 (reference)
**Per protocol**						
6EH (320)	18	2.03(1.09–2.97)	1.60(0.73–3.49)	25	2.82(1.72–3.93)	1.21(0.67–2.27)
36H (299)	11	1.30(0.53–2.07)	1.0 (reference)	20	2.37(1.33–3.41)	1.0 (reference)

**Table 3:** TB incidence and mortality rate among TST positive and negative subjects, by regimen (mITT population) and per protocol analysis.

mITT analysis						
		TB incidence/100 py (95%CI)	aIRR (95% CI)	Mortality/100 py (95%CI)	aIRR (95% CI)	
TST>5 mm	6EH (n = 141)	3.18 (1.38–4.97)	1.66 (0.63, 4.30)	2.91 (1.19–4.63)	1.51(0.56,4.02)	
	36H (n = 132)	1.81 (0.69–3.04)	Reference	2.53 (1.21–3.85)	Reference	
TST≤5 mm	6EH (n = 203)	1.94 (0.5–3.38)	1.48 (0.55, 3.96)	1.94 (0.5–3.38)	1.10 (0.50,2.41)	
	36H (n = 207)	1.23 (0.32–2.13)	Reference	2.28 (1.04–3.51)	Reference	
**Per protocol analysis**						
TST>5 mm	6EH (n = 131)	2.80 (1.06–4.70)	1.57 (0.50, 4.9)	3.08(1.26–4.89)		1.43 (0.53,3.8)
	36H (n = 116)	1.84(0.37–3.32)	Reference	2.15(0.56–3.74)		Reference
TST≤5 mm	6EH (n = 189)	1.52 (0.47–2.57)	1.51 (0.53, 4.3)	2.65(1.26–4.04)		1.04 (0.48, 2.29)
	36H (n = 183)	0.96 (0.12–1.81)	Reference	2.51(1.14–3.87)		Reference

**Table 4:** Crude TB incidence and death rates and adjusted incidence rate ratios: stratified analysis on m-ITT population.

	TB		Death	
	Cruderate/100 py (95% CI)	aIRR (95% CI)	Crude rate/100py (95% CI)	aIRR (95% CI)
Male	2·35 (1·12–3·58)	1·3 (0·64–2·59)	5·20 (3·37–7·03)	3·7 (1·87–7·15)
Female	1·31 (0·65–1·97)	1.0 (reference)	1·22 (0·58–1·86)	1.0 (reference)
CD4≤200 cells/mm^3^	4·07 (2·07, 6·06)	4·8 (2·29–9·85)	5·34 (3·05, 7·62)	2·9 (1·54–5·76)
CD4>200 cells/mm^3^	0·97 (0·44, 1·50)	1.0 (reference)	1·80 (1·08, 2·51)	1.0 (reference)

Patients who developed TB did so at a median of 12 months - overall, 34% of cases occurred in the first six months, 46% in the next twelve months and 20% thereafter. Fifteen patients had bacteriologically confirmed TB (10 in the 6EH arm and 5 in the 36H arm), most had some symptoms (cough, fever or malaise) at the time of TB breakdown (supplementary appendix-2).

The incidence of bacteriologically confirmed TB among TST positive individuals in 6EH regimen and 36H regimen was 1.9/100py (1.8–12.2) and 0.8/100py (0.62–8.8), with the incidence rate ratio being 1.54 (95% CI 0.19–2.88) p = 0.63. Of the 15 culture-confirmed cases, 8 had *M.tuberculosis* susceptible to all first-line drugs, 4 were resistant to isoniazid, 1 to streptomycin, 1 to isoniazid and rifampicin and 1 to streptomycin, isoniazid and rifampicin. In 20 patients (12 in the 6EH arm and 8 in the 36H arm) the diagnosis of TB was based on clinical, radiographic or histopathologic evidence (supplementary appendix 3).

**Figure 2:**
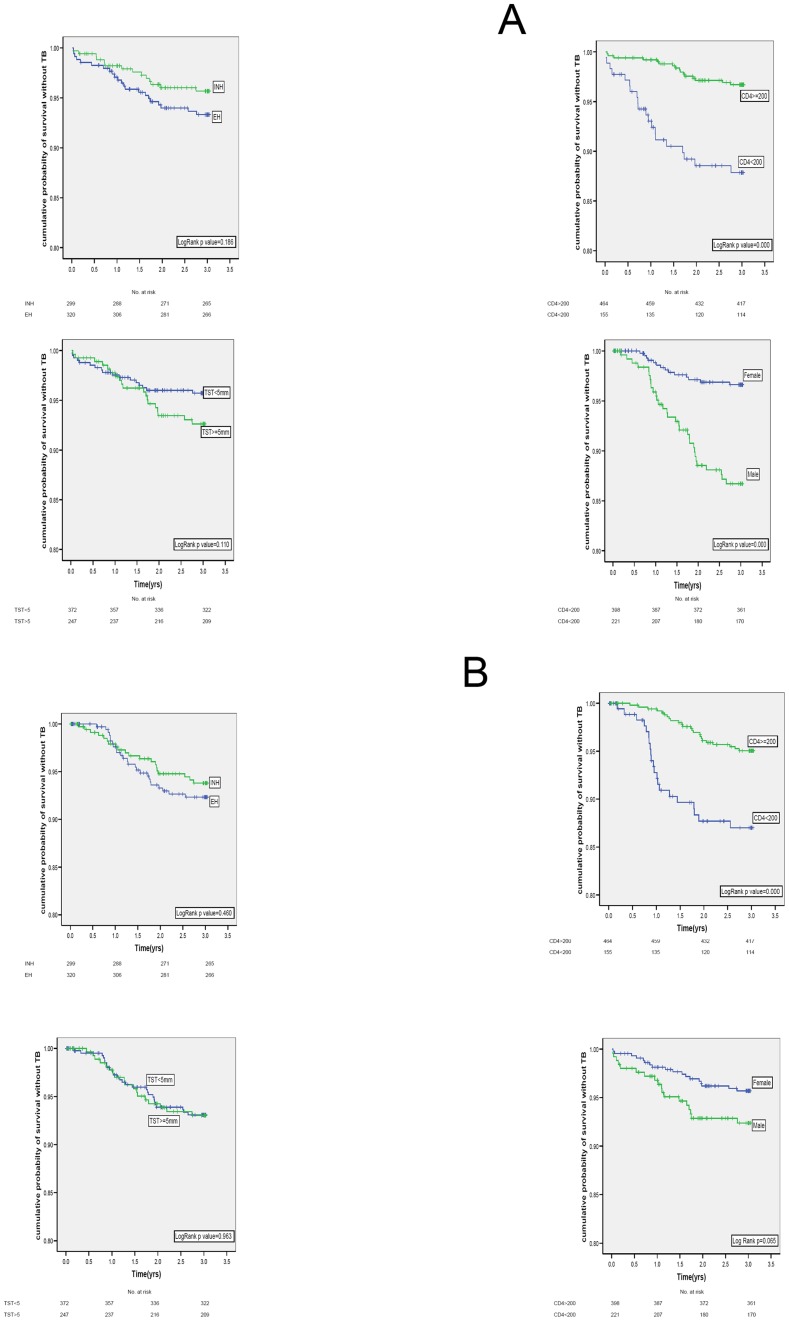
Kaplan-Meier curves showing cumulative probability of survival without TB and death over 36 months by regimen, TST status, CD4 count and sex. The four top panels show cumulative survival without TB over 36 months by regimen (6EH versus 36H, ns), TST (<5 mm versus >5 mm, ns), CD4 count (<200 versus >200 cells/mm3, p<0.001) and sex (female versus male, p = 0.05). The four bottom panels show cumulative mortality over 36 months by regimen (6EH versus 36H, ns), TST (<5 mm versus >5 mm, ns), CD4 count (<200 versus >200 cells/mm3, p<0.001) and sex (female versus male, p<0.001).

Twenty-five patients in the 6EH arm (2·8/100PY, 95%CI 1·7–3·9) and 20 patients in the 36H arm (2·2/100PY, 95%CI 1·2–3·2) died during the 36-months. There was no difference in mortality by regimen. Death rate was significantly higher among males and among patients with CD4 count below 200 cells/mm^3^ but similar in individuals with positive and negative TST status (Figure 2A & 2B, Tables 3 & 4). The median CD4 at the time of death was 66 (IQR 36–132) and 73 (IQR 40–308) cells/mm^3^ in the 6EH and 36H arms, respectively. Two patients in the 6EH arm had been initiated on ATT by the treating physician shortly before death and were therefore considered as “probable TB deaths”, though no confirmatory evidence was available. Since death was the first reported event, these patients have been included in the mortality analysis but not as incident TB cases. The most common cause of death was progressive HIV disease with complications/opportunistic infections (Table 5).

**Table 5:** Causes of death.

Cause of Death	6EH (n = 25)	36H (n = 20)
CNS complications of HIV	4	4
CVA (cerebral infarct)	2	1
Diarrhoea with wasting	4	3
Opportunistic Infections	3(Pneumonia-1, Cryptococcal meningitis-1, Disseminated *M. avium* disease-1)	3(Pneumonia-1, Pneumocystis jirovecci pneumonia -1, Disseminated Candidiasis -1)
AIDS-related complications	5	1
Others	5(Unknown-3, CA Lung-1, Suicide-1)	8(Unknown-2, Myocardial infarction-3, Road traffic accident-1,CA larynx-1, Suicide-1)
TB (probable)	2	0

Overall, the study drugs were well tolerated, with 3% of patients reporting grades I–IV toxicity relating to treatment (22 adverse events) (Table 6). Three patients in each arm had increased bilirubin levels; drugs were temporarily withheld and successfully re-introduced after liver function tests returned to normal. Two patients in the 36H arm had severe peripheral neuropathy necessitating permanent withdrawal of isoniazid, one at the 23^rd^ month and the other at the 33^rd^ month.

**Table 6:** Adverse Drug Reactions (based on common toxicity criteria manual [22]).

	6EH (n = 344)			36H (n = 339)		
	GradeI/II	GradeIII	GradeIV	GradeI/II	GradeIII	GradeIV
Arthralgia	1	-	-	-	1	-
Nausea, vomiting gastritis	2	1	-	1	-	-
Jaundice	-	-	3#	-	2#	1#
Neuropathy	2	-	-	1	1	2*
Cutaneous	2	-	-	1	1#	-
Any ADR	11			11		

# - Drugs temporarily withheld and re-introduced (n = 7).

* Drug permanently discontinued (n = 2, one at 23rd month, one at 33rd month).

## Discussion

A six-month regimen of isoniazid and ethambutol (6EH) and a 36-month regimen of isoniazid alone (36H), considered as proxy for life-long therapy, were equally effective in preventing active TB among HIV-infected individuals in India. The incidence rates were substantially lower (by 65% and 78% respectively) than observed in a previous cohort study of HIV-infected patients in the same geographic area [8]. While TB incidence was approximately 40% lower with 36H than with the 6EH regimen, this difference was not statistically significant However, due to the lower than expected event rate in the trial, the power of the study to detect a difference of >5% cumulative TB incidence (or 1.6%/year) was 65%, limiting the strength of our conclusions. Results using per-protocol, ITT and modified intent-to-treat analysis were however consistent, suggesting that a difference between arms was not attenuated by decreased adherence in the 36H arm. Considering only confirmed TB, the 36H group had 5 cases versus 10 in 6EH, suggesting that the longer regimen could have been more effective, but the difference was not statistically significant. However, as TB in HIV-infected persons is often difficult to confirm by sputum culture, clinically diagnosed cases also need to be considered for analysis for extrapolation into real life settings.

The issue of optimal duration of prophylactic regimen has recently been addressed in two studies. Similar to our study, Martinson et al showed that while there was a lower incidence rate of TB in their continuous Isoniazid arm, this was not statistically significant compared to the shorter regimen of 6 months of Isoniazid. [26]. In contrast, a study comparing six-months versus 36-months of IPT in Botswana reported a significantly greater efficacy with the longer regimen, an effect which was more pronounced among TST-positive individuals [27]. Major differences between the Botswana and this trial include a higher TB incidence, open labeled design, use of 1 TU PPD, lower power of the study (India) versus double-blind placebo controlled design, use of 5TU PPDRT23, larger number of TST+ individuals and greater use of ART in the Botswana study. These factors could potentially account for the contrasting results.

Both regimens in our study were well tolerated, with only two patients discontinuing therapy due to severe peripheral neuropathy (both in the longer regimen). In one meta-analysis, the efficacy of prophylactic regimens was similar irrespective of drug type, frequency or duration of treatment. However, short course multidrug regimens (especially with pyrazinamide) were much more likely to require discontinuation due to toxic effects [28].

Incidence rates of TB were not statistically significantly different in the two regimens when analysis was restricted to TST positive individuals, considering overall TB as well as bacteriologically confirmed cases. However TB rates in the 36H arm were lower compared to 6EH both among TST positive and negative subjects, suggesting that the longer regimen may protect both against exogenous infection as well as reactivation of latent disease [29]. Our previous findings that TST (using 1TU PPD) has poor sensitivity in detecting latent TB in patients with HIV suggest that the role of TST in screening patients for TB preventive therapy needs to be examined further perhaps using higher strengths of PPD [30].

The duration of protective effect of TB preventive therapy for HIV-infected patients ranges from 12 months to three years and appears to be higher with multi-drug therapy [12], [28], [31], [32]. In the current study also, the median time to develop TB among subjects in the 6EH arm was 12 months from the end of treatment.

Four patients developed isoniazid resistant TB while two had MDR-TB. This is consistent with the number of expected drug resistant incident TB cases (six for isoniazid and 1·6 for MDRTB) extrapolated from previous data in a similar population without exposure to TB preventive therapy (supplementary appendices 4a and 4b) [19]. Successful preventive treatment of isoniazid susceptible latent infections would leave mainly resistant infections to re-activate, suggesting that re-treatment regimens should be employed for patients who develop TB on IPT. Previous reports including one systematic review did not observe an increased risk of drug resistant TB after preventive therapy (risk ratio 1.45, 0.85–2.47), but numbers of patients in individual studies were small [33], [34].

In the current study, mortality rates were not statistically different between the two arms. Most patients died of complications of advanced AIDS before they could access ART. Retrospective observational studies in South Africa and Brazil have reported an additive benefit of IPT and ART [35], [36]. The intensive screening for TB that is part of the package of care recommended for patients initiating ART and treatment of active TB patients are other co-benefits of enrolment in an IPT program, likely to lead to mortality reductions [37]. Given the updated WHO recommendations for initiation of ART at a CD4 count of ≤350 cells/mm^3^, we anticipate that the efficacy of IPT will improve as patients gain wider access to ART.

Among the strengths of our study are high rates of adherence and follow-up due to intensive monitoring strategies in both therapy arms, thorough TB screening at routine intervals to minimize detection bias, and consistent results between the ITT, modified intention-to-treat and per-protocol analyses. Though ours was an open label trial, the Berger-Exner test showed that there was no unobservable selection bias. The results should be interpreted with caution, however, in light of certain limitations. Firstly, the impact of the ethambutol in the 6EH arm is not clearly understood, especially because of the lack of a 6H arm. The lack of ethambutol resistance in active TB cases and low risk of drug toxicity in both arms makes it an ideal companion drug. Further study will be required to assess the efficacy advantage of equal length TB preventive therapy regimens with isoniazid plus ethambutol over isoniazid alone. The absence of a concurrent placebo or control arm was substituted by the use of historical data from the same setting, though this is obviously not ideal. Thirdly, at completion, our trial was underpowered to find a difference in protective effect, especially in the analysis stratified by TST status. Finally, as noted above, limited access to ART in the first several years of the trial resulted in increased TB risk and mortality, possibly leading to underestimation of the benefit of TB preventive therapy.

The evidence from our trial suggests that both 6 and 36-month preventive therapy regimens are safe and highly effective (with a trend to higher efficacy with the longer regimen) in preventing TB among HIV-infected individuals in India. Policy makers can make a choice based on feasibility and other logistic considerations from a public health standpoint but it is clear that this intervention should be considered a priority for inclusion in the package of care and support for HIV-infected patients in TB-endemic countries.

## Supporting Information

Protocol S1 **Trial Protocol.**
(DOC)

Checklist S1 **Consort Checklist.**
(DOC)

Appendix S1 **Trial profile.**
(TIFF)

Appendix S2 **Details of patients who developed bacteriologically proven incident TB disease (n = 15).**
(DOC)

Appendix S3 **Details of patients who developed clinically diagnosed TB disease (n = 20).**
(DOCX)

Appendix S4 **(A) Two by two table demonstrating the likelihood of an isoniazid resistant strain of tuberculosis when no isoniazid prophylaxis is provided, among 100 patients with HIV-associated TB in India.** (B) Two by two table demonstrating the likelihood of an isoniazid resistant strain of tuberculosis when isoniazid-based prophylaxis is provided, among 100 patients with HIV-associated TB in India.
(DOCX)
