# Supplementary material for: Efficacy of a Six-Month versus a 36-Month Regimen for Prevention of Tuberculosis in HIV-Infected Persons in India: A Randomized Clinical Trial
Source: PLoS One. 2012 Dec 14;7(12):e47400. doi: 10.1371/journal.pone.0047400 (PMC3522661; doi:10.1371/journal.pone.0047400)
Supplement: Protocol S1 — Trial Protocol. (DOC) [file pone.0047400.s001.doc]

**Evaluation of efficacy of two different preventive therapy regimens for tuberculosis in HIV infected persons**

## INTRODUCTION

##### HIV and Tuberculosis

The HIV infection has increased the burden of TB, especially in populations where the prevalence of TB infection is high among young adults. India has the largest number of HIV-infected people, largest number of tuberculosis cases and also the largest number of dually infected individuals, in the world. Infection with HIV is the most potent known risk factor for progression to active tuberculosis among adults.

Tuberculosis hastens the development of AIDS in HIV-infected persons. Individuals who are not HIV-infected and who become infected with *Mycobacterium tuberculosis* have an approximately 10 percent lifetime risk of developing active tuberculosis, compared with a risk of 60 percent or more in persons infected with HIV*.* This is particularly important in India where it is estimated that more than half of the adult population harbors *M.tuberculosis.*

Current estimates indicate that TB is by far the commonest opportunistic infection occurring among HIV positive persons in India and that 60 to 70% of HIV positive patients will develop tuberculosis in their lifetime. Several studies from different parts of the country have also documented high HIV sero-prevalence rates among tuberculosis patients. In a developing country like India, the potential extra burden of new tuberculosis cases attributable to HIV could overwhelm the already stretched tuberculosis budgets and support services.

Despite their increased susceptibility to TB, patients with HIV/AIDS can be cured of TB by Directly Observed Therapy, Short-Course (DOTS). DOTS is a technical and management package aimed at diagnosing and curing as many infectious tuberculosis cases as possible with the twin goals of >85% cure rate and 70% case-finding. If effectively implemented, this strategy should cut the chain of transmission in the community by curing most of the infectious cases. The Revised National Tuberculosis Control Program (RNTCP) utilizes the five components of DOTS and is being implemented in India in a phased manner from 1993 onwards.To date, a population of 250 million has been covered and the entire country is expected to be covered by 2004. Recognizing the serious threat posed by TB/ HIV co-infection, the RNTCP calls for strengthening collaboration between TB and AIDS control programs for better management of HIV-infected patients with TB. However, to date, there are no specific technical guidelines for diagnosis, treatment or prevention of TB among HIV infected persons.

Diagnosis of tuberculosis among HIV-infected persons is often delayed and difficult to make, as HIV-infected persons with TB are more likely to have negative sputum smears and minor and non-specific x-ray abnormalities. Among HIV-infected persons with an intact immune system, the clinical presentation is similar to that among HIV negative individuals. However, the pathogenesis and clinical features of TB change markedly among HIV positive persons with immunodeficiency (e.g CD4 counts of < 200). Studies comparing the proportion of sputum smear positive patients among HIV infected and non-infected tuberculosis patients have found a lower proportion of sputum smear positive patients among those with HIV infection compared with HIV negative patients. Recently, a diagnostic algorithm using the response to a course of broad - spectrum antibiotics has been used in an attempt to improve the specificity of diagnosis of smear-negative tuberculosis and this approach has been incorporated into the RNTCP also.

Among HIV-infected persons there is a prime need for early and accurate detection of smear positive TB to prevent further spread in the community, and there is also a need to accurately diagnose and treat smear-negative cases not only for individual health benefits but also to reduce the number of smear-positive cases that arise in future. It is unknown whether the current diagnostic criteria and investigations recommended by the RNTCP are adequate to effectively diagnose tuberculosis in HIV positive persons. Specifically, the use of less stringent or broader symptom complex for screening and the yield from 3 additional sputum specimens for AFB smear and mycobacterial culture needs investigation.

Although optimal treatment of TB among HIV-infected persons is crucial, current knowledge and recommendations for duration of treatment in this group of patients are equivocal. In the past decade several studies have examined the effectiveness of treatment of TB among HIV-infected persons (Annex 1). A study has also suggested that post-treatment maintenance therapy with a combination of Isoniazid and Sulfadoxine-Pyrimethamine significantly reduces recurrence rates and decreases mortality following short-course chemotherapy. Recently the effect of Trimethoprim-Sulfamethoxazole (Co-trimoxazole) in significantly decreasing mortality and hospital admission rates in HIV-infected patients with tuberculosis has also been demonstrated.

In India, the Revised National Tuberculosis Control Program (RNTCP) is being introduced in a phased manner and it is expected that all tuberculosis patients will be treated with intermittent short-course regimens using the DOTS strategy.Under the RNTCP, HIV/TB patients are currently being treated with the standard regimens, but the effectiveness of these regimens among TB patients with HIV infection is unknown.

Preventive therapy (PT) against TB is the use of one or more anti-tuberculosis drugs given to patients with latent infection with *Mycobacterium tuberculosis* in order to prevent the progression to active disease. HIV is the major cause of the large increase over the last decade in the incidence of tuberculosis in populations with a high prevalence of HIV infection. Several studies have now demonstrated that PT is effective in preventing TB in individuals dually infected with HIV and *M. tuberculosis*. Studies conducted in USA, Africa and Haiti indicate that preventive therapy is efficacious in HIV positive persons with tuberculin reactions > 5mm, and that the optimal duration of isoniazid preventive therapy (using a single drug) should be greater than six months to provide the maximum degree of protection. Recently, a two-month regimen of daily rifampicin and pyrazinamide was found to provide protection equivalent to daily isoniazid for one year. The advantage of shorter regimens is better patient compliance and possibly earlier sterilization of lesions; however, the risk of drug toxicity maybe higher. The efficacy of preventive therapy in Mantoux negative HIV+ persons has not been clearly established, most studies showing it to be of limited value. Results of prevention trials conducted around the world are summarized in Annex 2.

Although there is large body of literature indicating benefit from preventive therapy, particularly among Mantoux Test (MT) positive persons, several issues regarding preventive therapy for tuberculosis in HIV positive persons need to be addressed in the endemic Indian setting. Due to the continuous environmental exposure to mycobacteria in the Indian setting, it is likely that HIV positive persons are at increased risk for disease due to both re-activation and re-infection. Hence, HIV positive persons who are Mantoux negative could also be at a high risk for developing tuberculosis following primary infection. In this setting, there is need to evaluate the efficacy of prophylaxis for tuberculosis in both tuberculin positive and negative individuals. The duration of preventive therapy required in an endemic setting is another areas requiring further investigation. Currently, preventive therapy is not included as part of the RNTCP. However, the growing problem of HIV in India could make prevention of tuberculosis in this high-risk group a priority area in the years to come.

**Purpose of the proposed study**

The HIV epidemic has a major impact on the epidemiologic dynamics of tuberculosis and poses several challenges to control of TB in a resource-poor country like India. HIV/TB burden can be brought under control by interventions that reduce the risk of progression of TB infection to overt disease (by preventive therapy), by timely diagnosis of TB among HIV-infected by cases finding using enhanced diagnostic criteria, and by adequate and effective treatment. Given the serious threat posed by the HIV epidemic on control of TB, and the current gaps in knowledge related to diagnosis, prevention and treatment of TB among HIV-infected persons in the Indian population, it is proposed to conduct a randomized control clinical study with the following interrelated objectives.

**Study Objective**

To compare the efficacy of two TB preventive therapy regimens in reducing the incidence of tuberculosis and mortality among HIV-infected persons.

**Study Design**

The study will be a two-armed prospective randomized clinical trial among HIV- positive patients without active tuberculosis. Enrolled patients will be assigned to one of the two unsupervised self-administered treatment regimens i.e. EH for 6 months or INH alone for 3 years. At the end of a 3-year follow-up, incidence of TB and overall mortality will be compared in each group.

**Study population and enrollment**

All HIV positive patients seeking care at one of the study centers, above the age of 15 years, not suffering from a serious illness, non-pregnant, and in whom TB was ruled out using the enhanced screening process described in section I, will be approached to participate in the study. The consent form for prophylaxis trial will be read to the patients in the local language and written consent will be obtained from willing patients.

**Enrollment and baseline investigations**

All patients who provide a written consent to participate in the trial will be interviewed by a social worker using a standard questionnaire. Patients with previous history of TB treatment of greater than 1 month or those on anti-retroviral drugs will be excluded from the study. Blood samples will be collected from patients meeting initial eligibility criteria to test for laboratory eligibility criteria. Standardized laboratory investigations will include complete heamogram (total and differential leucocyte counts, platelet counts and haemoglobin estimation), Renal function test (serum uric acid, blood urea, serum creatinine), Liver function tests, random blood sugar (BS), urine albumin and urine sugar. Patients who fulfill laboratory criteria (hemoglobin =>70 g/L, granulocyte count >1.1 X 10^9^/L, platelet count > 100X 10^9^/L, serum alanine amino transferase concentration <2.5 times the upper limit of normal, and serum creatinine concentration <1.1 mg%, random blood sugar < 140 mg%) will be enrolled in to the study.

Patients whose CD4+ cell count is below 100 absolute count or 10% of the total leucocyte count will not be admitted to the study, as they may have a latent focus of tuberculosis, which cannot be diagnosed by standard techniques available.

**Sample Size**

Assuming that prophylaxis will result in reduction of the incidence of TB in HIV positive persons from 7/100 person years (published TRC study) to 3/100 person years, the sample size for each arm is calculated to be 250. Allowing for a 10% drop out rate per year due to death or default, the number of patients required per treatment arm will be 325.

Total number of patients to be admitted to the study: 650

**Randomization and Dosing**

All patients enrolled in the prevention trial will be randomized to one of the study groups using a permuted block randomization scheme of four. Stratification will be done by Mantoux reading (>5mm and ≤5mm). The group assignment list will be generated centrally before the start of trial and sequentially numbered sealed envelopes containing the study group assigned will be prepared independently. At the initiation of prophylaxis, each patient will be counseled about the importance of taking drugs regularly. Patients will be asked to return the empty packets as well as unused tablets at each monthly visit.

# The treatment regimens in each study group will be as follows:

1. Ethambutol (800 mg) and Isoniazid (300 mg) daily for six months, self-administered, collected once in fifteen days.
2. Isoniazid (300 mg) daily for 3 years (in lieu of lifelong prophylaxis) with fortnightly collection of drugs

Subjects in both study groups will receive 10 mg of Pyridoxine daily during treatment.

**Follow-up**

During chemoprophylaxis

All enrolled patients will be followed up regularly to monitor general clinical status, adverse effects, adherence to drugs and development of active tuberculosis. Patients will be called to the clinic three monthly for a thorough clinical assessment and measurement of weight (see table). During each clinic visit, patients will be verbally screened for chest symptoms. In the presence of chest symptoms, the patient will be thoroughly evaluated clinically, and investigated for tuberculosis by radiological examination and 3 sputum specimens for smear and culture.

During each follow up visit the patient will asked for any symptoms or signs of drug toxicity. Details of any adverse reactions and their management will be recorded on a standardized toxicity form. Adverse reactions will be appropriately managed.

Adherence to the assigned regimen will be checked by detailed questioning of patients, return of empty drug packs, and by examination of spot urine samples for acetyl isoniazid. Non-adherent patients will be counseled regarding importance of treatment regularity and monitored closely. If a patient fails to attend the on the due date for drug collection, a letter will be posted, following which a health visitor or social worker will visit his/her home in case there is no response within a week.

Post Chemoprophylaxis

Patients will be followed up for a period of three years from the time of admission to the study. Clinical examination and relevant investigations will be done 3 monthly. Patients suspected to have tuberculosis at any time will be fully investigated and treated appropriately. Any positive culture will be subjected to drug susceptibility tests. If a patient dies, a verbal autopsy will be conducted to ascertain the cause of death**.**

## Investigation and follow up

| Clinical Evaluation | Sputum smear and culture | Mantoux Test | Urine for Alb, Sugar, Ac-INH | Hemogram, Renal & Liver function test | Chest  X-ray | CD4/CD8 Viral load, Immunol. Investigns. |
| --- | --- | --- | --- | --- | --- | --- |
| 0 month & every 3^rd^ month till completion of study, and whenever patient is symptomatic | 0 month (3 specimens), 6,12,18,24, 30,36 mths (2 spec) and if ordered by doctor | 0 month and at clinical diagnosis of TB | 0 month, every month till 36 months | 0 month & if any toxicity | 0 month, 6,  12, 18, 24, 30, 36 months and if ordered by doctor when patient is symptomatic | 0 month, 6, 12, 18, 24, 30 and 36 months |

### Radiographic and/or Clinical Deterioration:

a) If a patient has been admitted to the preventive therapy study and baseline sputum culture/s are reported as positive, the patient should be called up to the clinic, re-evaluated and started on chemotherapy (Category I or II depending on duration of treatment). If the patient is not suitable for Study XX, then he/she can be treated in Zero study – adequate treatment for these patients must be ensured.

b) If during the treatment period, the patient becomes symptomatic, clinical examination will be done, an X-ray ordered and 3 sputa collected for smear and culture. A course of antibiotics will be prescribed and a repeat chest X-ray done after 15 days. If the symptoms are not respiratory in origin, then evaluation and referral to the appropriate department for further investigations will be done. All attempts will be made to make a definite diagnosis including investigations like pleural tap, lymph node biopsy, ultrasound abdomen and CSF examination where necessary. The treating doctor, in consultation with TRC clinic physicians, will decide about the need for anti-tuberculosis treatment. The case will be put up to the Weekly Panel for a decision. Normally, patients diagnosed to have tuberculosis will be admitted to the Chemotherapy study (if eligible and suitable) and treated with Category II. Modifications may be made after the culture and sensitivity results are available.

**Statistical analysis and outcome measures**

The primary end point of the study will be development of tuberculosis and the secondary endpoints will include adverse drug reactions and mortality rate. The intention-to-treat approach will be used for analyzing the data for primary and secondary end points. Annual interim analyses will be done to ensure timely identification of any significant risks or benefits to patients. The treatment groups will be compared to note random distribution of baseline demographic and socio-economic, clinical and laboratory characteristics. The incidence of tuberculosis will be estimated by the person-year method. Cumulative proportion will be used for adverse reactions and mortality. The log rank test of significance will be used to compare cumulative incidence of TB in the treatment groups. Survival analyses using the log rank testing the null hypothesis and Kaplan Meier curves for depicting cumulative survival in TB and mortality will be done to compare outcomes between study groups. Multivariate analyses will be performed using Cox’s proportional Hazards model.

**ETHICAL ISSUES**

The study design includes several features to avoid any potential harm to the participants.

All patients approached for voluntary HIV testing will be provided pre-test counseling by a trained social worker. Written consent will be obtained from all participants before HIV testing. Following a positive result, post-test counseling will be done. Results of HIV test will be conveyed only to the subject and will be kept strictly confidential. Separate forms will be used to obtain informed written consent for participation in treatment or prevention trial. The consent forms will clearly explain the purpose of the study (chemotherapy or preventive therapy), overall benefits of the study to the patient and to the community, risk and benefit of laboratory investigations and drugs administered during treatment. Patient will also be informed about the number and frequency of clinic visits required, as well as home visits be made by clinic staff.

To ensure that participation in the study does not lead to any economic losses for the patient, social workers will make case-by-case evaluation and patients will be monetarily compensated for costs incurred due to travel, as well as due to wages lost, in case the patient is employed.

All seriously ill patients will be referred to the appropriate clinic and excluded from the study. Similarly, patients with laboratory abnormalities will also be excluded from the study and referred for medical management. The interventions used in the study include non-invasive tests that involve minimal risk to the participants. The risk benefit of drugs and procedures used in the study is weighted toward benefit. All patients under treatment will be regularly monitored to note any side effects or toxicity. In case of severe reactions the assigned treatment will be terminated and the patient will be appropriately management. Annual interim analysis will be performed to identify and closely monitor any risks or benefits to the patients.

The study protocol has been submitted for review and cleared by the Institutional Ethical Committees of TRC.

Wilkinson D, Squire SB, Garner P. Effect of preventive treatment of tuberculosis in adults infected with HIV: systematic review of randomized placebo controlled clinical trials. BMJ 1998; 317, 625-629.

Hawken MP, Meme HK, Elliot LC et al. Isoniazid preventive therapy for tuberculosis in HIV-1 inected adults: results of a andomized controlled trial. AIDS 1997; 11: 875-882.

Whalen CC, Johnson JL, Okwera et al. A trial of three regimens to prevent tuberclosis in Ugandan adults infected with the human immunodeficiency virus. N Engl J Med 1997; 337, 801-808.

Gordin F, Chaisson RE, Matts JP. Rifampicin and pyrazinamide versus isoniazid for prevention of tuberculosis in HIV infected persons: an international randomized trial. Terry Beirn Community Programmes for Clinical Research in AIDS, the Adult AIDS Clinical Trial group, the Pan American Health organization and the Center for Disease Control and Prevention Study Group. JAMA 2000; 283: 1445-1450

Grant AD, Djomand G, De Cock KM. Natural history and spectrum of disease in patients with HIV/AIDS in Africa. AIDS 1997; 11 (Suppl B): S43-54.

Swaminathan S, Ramachandran R, Baskaran G, Paramasivan CN, Ramanathan U, Venkatesan P, Prabhakar R, Datta M. Risk of development of tuberculosis in HIV-infected patients. Int J Tuberc Lung Dis 2000; 4(9):839-44.
