# Supplementary figures and images for: Efficacy of a Six-Month versus a 36-Month Regimen for Prevention of Tuberculosis in HIV-Infected Persons in India: A Randomized Clinical Trial
Source: PLoS One. 2012 Dec 14;7(12):e47400. doi: 10.1371/journal.pone.0047400 (PMC3522661; doi:10.1371/journal.pone.0047400)

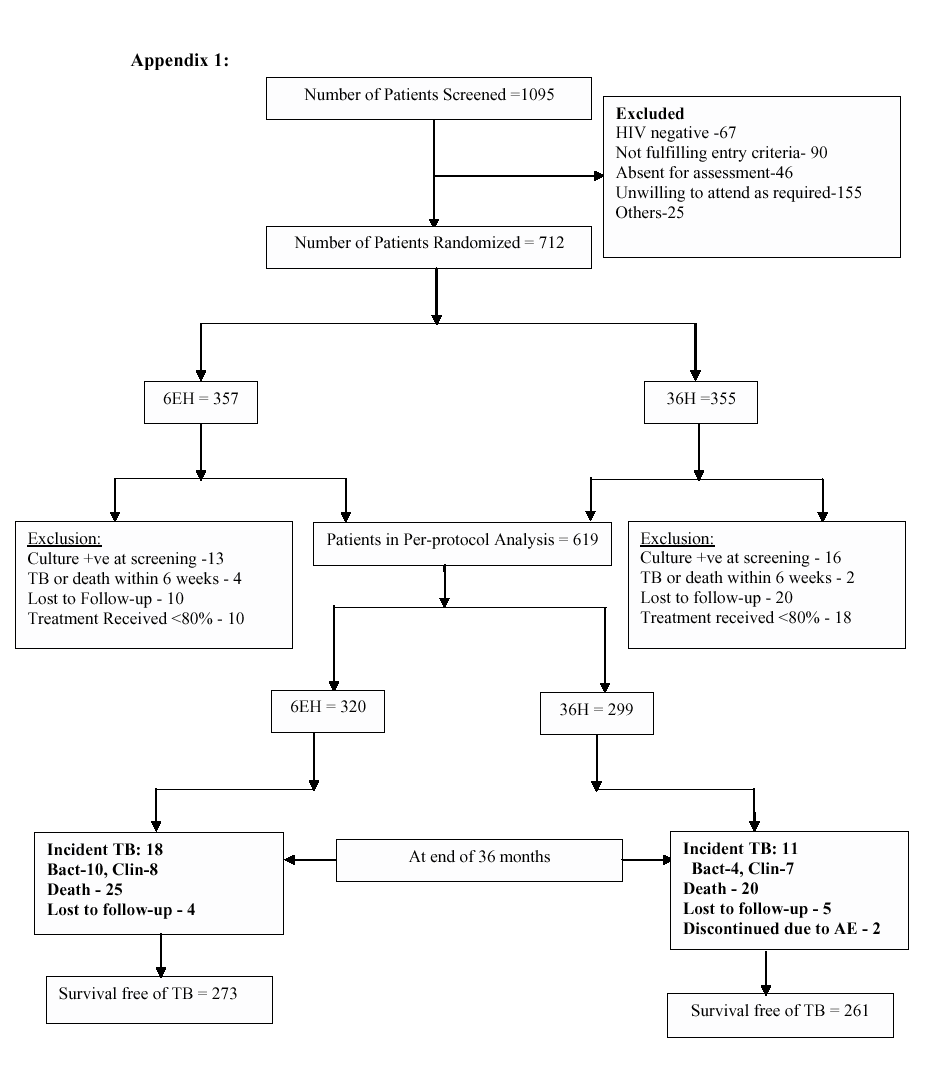

Supplement: Appendix S1 — Trial profile. (TIFF) [file pone.0047400.s003.tiff]
