# Supplementary material for: Efficacy of a Six-Month versus a 36-Month Regimen for Prevention of Tuberculosis in HIV-Infected Persons in India: A Randomized Clinical Trial
Source: PLoS One. 2012 Dec 14;7(12):e47400. doi: 10.1371/journal.pone.0047400 (PMC3522661; doi:10.1371/journal.pone.0047400)
Supplement: Appendix S2 — Details of patients who developed bacteriologically proven incident TB disease (n = 15). (DOC) [file pone.0047400.s004.doc]

Appendix 2: Details of patients who developed bacteriologically proven incident TB disease (n = 15)

| S. No. | Regimen | Age,  years | Sex | Month of TB break down | Drug Resistance | TST, mm | Baseline CD4, cells/mm^3^ | CD4 at Event, cells/mm^3^ | Symptoms at breakdown | Outcome of TB Treatment |
| --- | --- | --- | --- | --- | --- | --- | --- | --- | --- | --- |
| 1 | 36H | 39 | M | 12 | Nil | 17 | 62 | 38 | NA | Died |
| **2** | **36H** | **26** | **M** | **1** | **HR** | **3** | **38** | **30** | **Fever** | **Died** |
| 3 | 36H | 27 | F | 21 | SHR | 0 | 341 | 92 | Fever | Cured |
| 4 | 36H | 35 | M | 18 | S | 25 | 378 | NA | Cough | Cured |
| 5 | 36H | 26 | F | 24 | Nil | 14 | 132 | 51 | NA | Died |
| 6 | 6EH | 25 | F | 18 | Nil | 2 | 336 | NA | Malaise | Cured |
| 7 | 6EH | 28 | M | 12 | Nil | 20 | 42 | 40 | Fever | Died |
| 8 | 6EH | 26 | M | 20 | H | 6 | 189 | 130 | Fever | Cured |
| 9 | 6EH | 25 | M | 33 | H | 4 | 70 | 190 | Cough, breathlessness | Defaulted |
| 10 | 6EH | 35 | F | 23 | Nil | 10 | 570 | 306 | Cough, breathlessness | Cured |
| 11 | 6EH | 24 | F | 12 | H | 32 | 598 | 224 | Fever | Cured |
| 12 | 6EH | 28 | M | 18 | Nil | 26 | 144 | 207 | Fever | Cured |
| 13 | 6EH | 39 | F | 8 | H | 0 | 117 | NA | NA | Died |
| 14 | 6EH | 25 | F | 10 | Nil | 15 | 223 | 66 | Lymphadenitis | Cured |
| 15 | 6EH | 24 | F | 32 | Nil | 20 | 836 | 662 | Cough | Cured |

The highlighted row represents patients who developed TB in the first 6 weeks who have been excluded from per-protocol analysis
