# Supplementary material for: Efficacy of a Six-Month versus a 36-Month Regimen for Prevention of Tuberculosis in HIV-Infected Persons in India: A Randomized Clinical Trial
Source: PLoS One. 2012 Dec 14;7(12):e47400. doi: 10.1371/journal.pone.0047400 (PMC3522661; doi:10.1371/journal.pone.0047400)
Supplement: Appendix S3 — Details of patients who developed clinically diagnosed TB disease (n = 20). (DOCX) [file pone.0047400.s005.docx]

Appendix 3: Details of patients who developed clinically diagnosed TB disease (n =20)

| S. No. | Regimen | Age,  years | Sex | Month | Site of TB lesion | TST, mm | Baseline CD4, cells/mm^3^ | CD4 at event, cells/mm^3^ | Outcome of TB Treatment |
| --- | --- | --- | --- | --- | --- | --- | --- | --- | --- |
| 1 | 36H | 25 | F | 6 | Pulmonary | 3 | 77 | 84 | Died |
| 2 | 36H | 35 | M | 6 | Pulmonary | 6 | 44 | 32 | Died |
| 3 | 36H | 38 | M | 6 | Pulmonary with osteomyelitis elbow | 20 | 24 | 77 | Died |
| 4 | 36H | 24 | M | 18 | Pulmonary | 18 | 552 | 112 | Died |
| **5** | **36H** | **30** | **M** | **1** | **Pulmonary** | **0** | **80** | **80** | **Completed** |
| 6 | 36H | 30 | M | 6 | Pulmonary with CNS involvement | 0 | 48 |  | Died |
| 7 | 36H | 24 | M | 17 | Lymphadenitis –cervical | 3 | 416 | 341 | Completed |
| 8 | 36H | 40 | F | 14 | Pericardial effusion | 0 | 96 | 174 | Completed |
| 9 | 6EH | 25 | F | 12 | Abdominal | 16 | 189 | 32 | Died |
| **10** | **6EH** | **38** | **M** | **1** | **Pulmonary, abdominal** | **7** | **55** | **55** | **Died** |
| **11** | **6EH** | **38** | **M** | **1** | **Pulmonary** | **20** | **264** | **264** | **Died** |
| **12** | **6EH** | **38** | **M** | **1** | **Pulmonary** | **0** | **45** | **45** | **Died** |
| 13 | 6EH | 32 | M | 18 | Polyserositis (Abdominal, Pleura, meningitis) | 4 | 538 | 562 | Died |
| 14 | 6EH | 38 | M | 9 | Pulmonary | 2 | 52 | 15 | Died |
| 15 | 6EH | 26 | F | 24 | Abdominal, Pulmonary | 22 | 252 | 209 | Died |
| 16 | 6EH | 45 | M | 2 | Pulmonary, CNS | 0 | NA | NA | Died |
| 17 | 6EH | 35 | F | 12 | Pulmonary, CNS | 0 | 54 | 8 | Died |
| 18 | 6EH | 28 | M | 13 | Mediastinal LN , abdominal | 22 | 283 | 227 | Completed |
| 19 | 6EH | 24 | F | 6 | Abdominal, Pulmonary | 0 | 95 | 25 | Died |
| **20** | **6EH** | **30** | **F** | **1** | **Abdominal** | **0** | **285** | **285** | **Died** |

The highlighted row represents patients who developed TB in the first 6 weeks who have been excluded from per-protocol analysis.
