# Supplementary material for: Efficacy of a Six-Month versus a 36-Month Regimen for Prevention of Tuberculosis in HIV-Infected Persons in India: A Randomized Clinical Trial
Source: PLoS One. 2012 Dec 14;7(12):e47400. doi: 10.1371/journal.pone.0047400 (PMC3522661; doi:10.1371/journal.pone.0047400)
Supplement: Appendix S4 — (A) Two by two table demonstrating the likelihood of an isoniazid resistant strain of tuberculosis when no isoniazid prophylaxis is provided, among 100 patients with HIV-associated TB in India. (B) Two by two table demonstrating the likelihood of an isoniazid resistant strain of tuberculosis when isoniazid-based prophylaxis is provided, among 100 patients with HIV-associated TB in India. (DOCX) [file pone.0047400.s006.docx]

**Appendix 4a:** Two by two table demonstrating the likelihood of an isoniazid resistant strain of tuberculosis when no isoniazid prophylaxis is provided, among 100 patients with HIV-associated TB in India

|  | **INH-R** | **INH-S** | **Total** |
| --- | --- | --- | --- |
| **TB** | **2** | **8** | **10** |
| **No TB** | **18** | **72** | **90** |
|  | **20** | **80** | **100** |

INH-R rate 20%

**Appendix 4b:** Two by two table demonstrating the likelihood of an isoniazid resistant strain of tuberculosis when isoniazid-based prophylaxis is provided, among 100 patients with HIV-associated TB in India.

|  | **INH-R** | **INH-S** | **Total** |
| --- | --- | --- | --- |
| **TB** | **2** | **3** | **5** |
| **No TB** | **18** | **77** | **95** |
|  | **20** | **80** | **100** |

INH-R rate 40%

Note: The table assumes a 50% efficacy of preventive therapy regimens containing isoniazid and a baseline isoniazid resistance prevalence of 20% among TB patients in India. Because most of the cases prevented would be among subjects with latent isoniazid susceptible TB, proportionately more incident cases would demonstrate isoniazid resistance (40% in this case).
